# Autophagy3D: a comprehensive autophagy structure database

**DOI:** 10.1093/database/baae088

**Published:** 2024-09-19

**Authors:** Neha, Jesu Castin, Saman Fatihi, Deepanshi Gahlot, Akanksha Arun, Lipi Thukral

**Affiliations:** Computational Structural Biology Lab, CSIR-Institute of Genomics and Integrative Biology, Mathura Road, New Delhi 110025, India; Computational Structural Biology Lab, CSIR-Institute of Genomics and Integrative Biology, Mathura Road, New Delhi 110025, India; Computational Structural Biology Lab, CSIR-Institute of Genomics and Integrative Biology, Mathura Road, New Delhi 110025, India; Academy of Scientific and Innovative Research (AcSIR), Ghaziabad 201002, India; Computational Structural Biology Lab, CSIR-Institute of Genomics and Integrative Biology, Mathura Road, New Delhi 110025, India; Academy of Scientific and Innovative Research (AcSIR), Ghaziabad 201002, India; Computational Structural Biology Lab, CSIR-Institute of Genomics and Integrative Biology, Mathura Road, New Delhi 110025, India; Academy of Scientific and Innovative Research (AcSIR), Ghaziabad 201002, India; Computational Structural Biology Lab, CSIR-Institute of Genomics and Integrative Biology, Mathura Road, New Delhi 110025, India; Academy of Scientific and Innovative Research (AcSIR), Ghaziabad 201002, India

## Abstract

Autophagy pathway plays a central role in cellular degradation. The proteins involved in the core autophagy process are mostly localised on membranes or interact indirectly with lipid-associated proteins. Therefore, progress in structure determination of ‘core autophagy proteins’ remained relatively limited. Recent paradigm shift in structural biology that includes cutting-edge cryo-EM technology and robust AI-based Alphafold2 predicted models has significantly increased data points in biology. Here, we developed Autophagy3D, a web-based resource that provides an efficient way to access data associated with 40 core human autophagic proteins (80322 structures), their protein-protein interactors and ortholog structures from various species. Autophagy3D also offers detailed visualizations of protein structures, and, hence deriving direct biological insights. The database significantly enhances access to information as full datasets are available for download. The Autophagy3D can be publicly accessed via https://autophagy3d.igib.res.in.

**Database URL**: https://autophagy3d.igib.res.in

## Introduction

**Figure 1.**
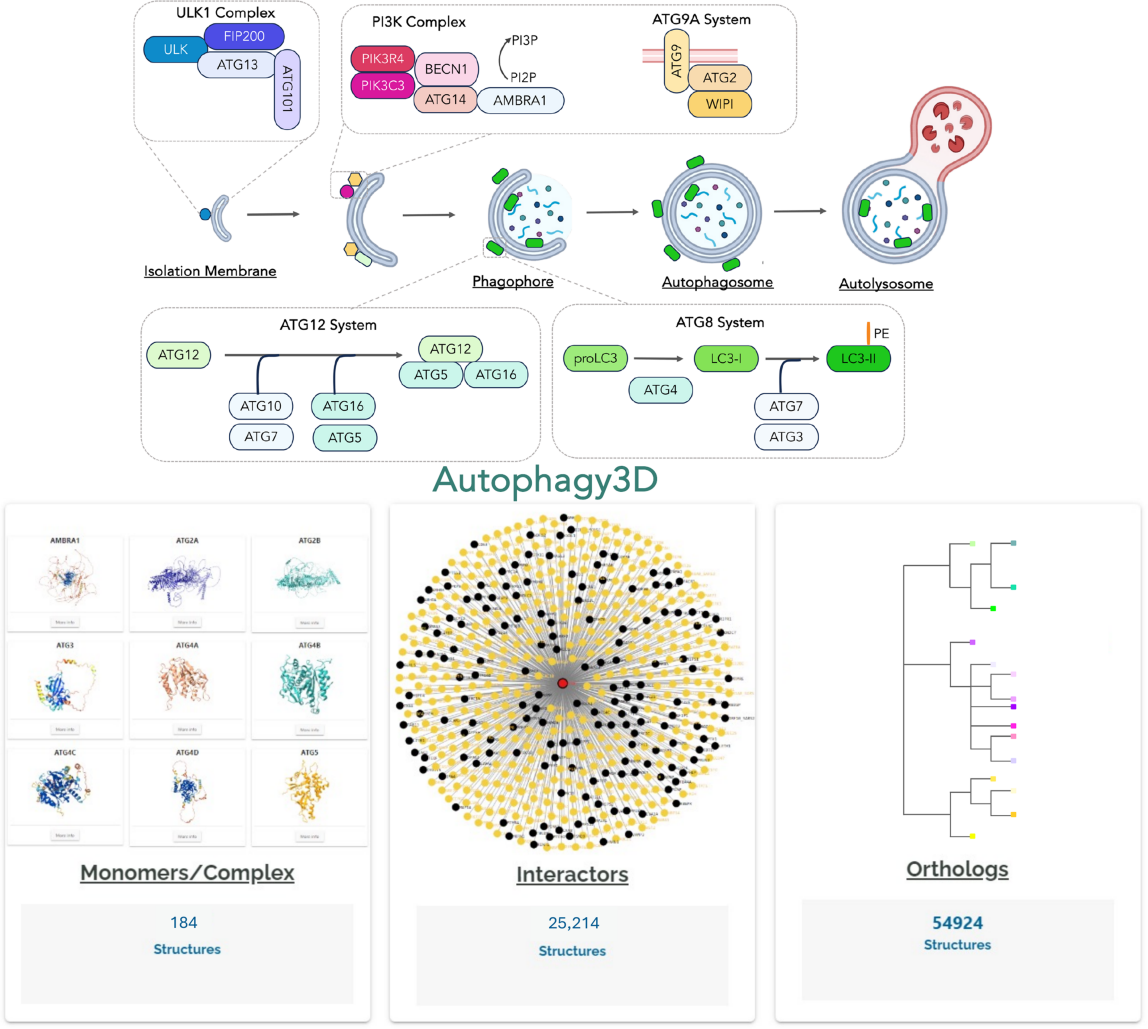
The overview of the Autophagy3D database provides comprehensive structural data points associated with 40 core autophagy proteins, information about their interactions orthologs.

Autophagy is a highly conserved eukaryotic process of cellular degradation. It involves the formation of a cup-shaped membrane that elongates and engulfs cytoplasmic content to form a double-membrane structure called autophagosome [1–3]. Autophagosomes are generated de novo in response to different stimuli such as starvation. Initially, an isolation membrane, also known as a phagophore, is generated, followed by its elongation and closure of the edge of the membranes to form autophagosomes. The entire process involves ∼40 core autophagic proteins (ATG proteins) that govern the formation of autophagosome which subsequently fuses with lysosome for the final degradation of enclosed cellular content [4–6] (Fig. 1a). The core ATG proteins are mainly divided into three different groups. Firstly, there is an initiation complex comprising of ULK1 and PI3K proteins, and then the ubiquitin machinery (E1, E2, E3-like complexes, and LC3/GABARAP family), followed by the lipid transfer complex (ATG9, ATG2). Under normal cellular conditions, ULK1 remains inactive due to its phosphorylation by the mTOR complex, which inhibits its kinase activity. However, when the cell encounters stress signals, ULK1 gets dephosphorylated, leading to its activation along with the associated proteins. The ULK1 complex comprises four key proteins: ULK1, ATG13, FIP200/RB1CC1, and ATG101 [7–11]. Upon activation, the ULK1 complex phosphorylates another essential autophagy complex, the PI3KC3 complex1 and PI3KC3 complex2, facilitating the generation of phosphatidylinositol-3-phosphates (PI3P) on the phagophore membrane [12, 13]. This step is critical in autophagosome biogenesis, as PI3P serves as a platform for downstream ATG proteins to recognize and bind to phagophore. The PI3KC3 complexes consist of PIK3C3 (lipid kinase), PIK3R4, BECLIN1, and ATG14 (in complex1) or UVRAG (in complex2) [14–16]. Simultaneously, the autophagy ubiquitin machinery is responsible for LC3/GABARAP family proteins lipidation onto the phagophore membrane. LC3 family proteins initially exist in a precursor form and undergo C-terminal cleavage by the protease ATG4 [17]. This lipidation involves a series of reactions mediated by E1-like (ATG7), E2-like (ATG10, ATG3) [18], and E3-like (ATG12-ATG5-ATG16) complexes [19], along with WIPI2 that binds specifically to PI3P lipids [20]. The WIPI family of proteins plays a crucial role in the early stages of autophagosome biogenesis, facilitating the recruitment of additional autophagy-related proteins [21]. A hallmark step of autophagy is also recruitment of LC3 family of proteins to the growing phagophore membrane [22, 23]. In humans, there are six homologs, including LC3A, LC3B, LC3C, GABARAP, GABARAPL1, GABARAPL2 [24, 25]. Despite sharing a common mechanism of action, LC3 and GABARAP proteins exhibit differences in their structure and interaction partners [26]. The growth of phagophore is mediated by ATG9-ATG2 complex. The ATG9, also known as lipid scramblase, is a multi-spanning transmembrane protein that primarily localizes to the Golgi apparatus [27, 28]. Overall, these ATG proteins directly govern autophagosome formation and, therefore, structural knowledge of these proteins is critical for our understanding in autophagy regulation.

Autophagy has recently gained attention as a key quality control process of cytoplasmic components and in particular, selective autophagy that involves the elimination of misfolded aggregates, damaged organelles (such as mitochondria, and peroxisomes) and invasive microbes [29]. Autophagy, in general, plays a role in pathological conditions like cancer, especially during the earlier stages of tumorigenesis [30]. Different strategies for modulation of autophagy are proposed for tumour selection or degradation of misfolded proteins [31]. Recent developments in autophagy-related drug delivery have revealed several small molecules that target autophagic machinery [32]. In addition, critical protein-protein interactions (PPIs) related to autophagy have also been reported for therapeutical potential [33]. Therefore, structural information on autophagic proteins is of high importance for the mechanistic and rational drug discovery process.

Several primary protein information resources, such as RCSB PDB (central repository for experimental structures) [34, 35] and UniProt (a universal knowledgebase for curated information related to proteins) [36, 37] have developed organised information on protein structure derived from experimental techniques. Recently, AI-based AlphaFold has provided robust predicted models with high-confidence score [38]. This has enabled the integration of AlphaFold2 database [39] with several primary data sources [9], and several new data sources deriving insights from it [40–44]. Our recent data suggests that AlphaFold2 significantly expands the structural space of the autophagy pathway and key autophagic proteins having low homology with existing experimentally resolved proteins are available as high-confidence models [45]. In addition, more than 214 million protein structure predictions are made available for retrieval, encompassing the proteome data from 48 organisms [39]. This information can also assist in understanding fundamental question ns related to the evolution of proteins, and their interactions. Thus, novel structural principles can be derived from them. However, structural models predicted by AlphaFold2 (AF2) need to be studied with caution due to several associated limitations. Some of the reported challenges include lower confidence in uncharacterised regions [46], inconsistent model with multiple protein states [47, 48], relative domain orientation [49], and novel protein folds that are often under-represented in the known population. Additionally, AlphaFold is prone to hallucinations, inherently predicting certain segments as helices which is prevented in AF3 by cross-distillation [50].

Given that the structural repository targeted to autophagy proteins is largely missing, we developed Autophagy3D which is a web-based platform for exploring structures of human autophagy core proteins. Three main categories (related to autophagy proteins) that have been extensively curated include structures of the human core autophagic proteins, their interactors, and ortholog representation of each species. It offers visualization of structures and comprehensive details of sequence, interactors, and orthologs. Several specialized structural resources (for proteins such as kinases) in the context of important biological pathways are available [51–53]. Although relevant databases which offer curated information related to autophagy proteins [54], regulatory networks [55] and expression profiles of autophagic proteins [56] are available, no user-friendly/interactive resource has been curated collectively for structures so far. Overall, Autophagy3D serves as a one-place resource for autophagy proteins and offers valuable resource on structures through interactive browser.

## Materials and methods

### Data collection

For the development of the database, 40 core human autophagic proteins that are known to have critical functions in different stages of autophagy have been considered. Three major structural data points associated with the human core autophagic proteins, their interactors and extensive orthologs were collected. Below we describe how each dataset was obtained.

#### Core structures

The information regarding the experimental and AlphaFold structural availability of the core proteins was retrieved from UniProt [34–37]. Later, all experimental structural data (coordinate files) and their corresponding meta-information such as the resolution (in case of X-ray structures), chain information, experimental method used, and presence of any non-protein entities such as small molecules and ions were obtained from Protein Data Bank [34, 37]. The experimental structure coverage was calculated for each PDB entry belonging to the core protein. For each core protein, the experimental structure with maximum coverage was considered for calculating the percentage of autophagic proteins with substantial coverage. In case the experimental structure was unavailable or the structural coverage was less than 70%, the corresponding AlphaFold structure was considered. Out of the total, 92 experimental structures had structural coverage of more than 90%. In addition, each PDB entry of core autophagic proteins was checked if the respective protein of interest was available in monomeric form or as a part of the multimeric complex. The complexes were later categorized into subcategories which include homo-oligomers, hetero-oligomers, peptide-bound and small-molecule bound. The LC3 proteins bound/fused with LC3 interacting regions (LIRs) were considered as peptide-bound. As of January 2024, we could obtain around 184 experimental structures. Out of them, 33 structures have been captured in monomeric form while 73 structures are categorized as hetero-oligomers, 4 structures as homo-oligomers, 95 structures as peptide-bound, and 33 structures as small molecule bound. For these proteins, 40 AlphaFold models are available. Out of them, 32 models have an overall pLDDT score of more than 70.

#### Interactors of human core autophagic proteins

The interactors for autophagic proteins were extracted from the BioGrid 4.4 database. BioGrid offers interactions that are supported by at least one experimental study [57]. The data on the structural availability for the interactors were fetched from UniProt ID and corresponding structures were downloaded from PDB. In the absence of structural information, the AlphaFold predicted model was considered. On an overall scale, 6377 interactors were retrieved and curated through ID mapping. Out of the total interactors, 6374 interactors were mapped to UniProt for secondary annotation. We retrieved experimental structures for 4491 interactors and AF-predicted models for 6227 proteins. For 4491 interactors (which have experimental structures), 25 214 PDB entries are present.

#### Ortholog structures

For each core autophagic protein, its respective orthologs were retrieved from OrthoDB [58]. For each NCBI GeneID of core human autophagic protein as query, we obtained the corresponding sequence hits. The resulting sequences were preprocessed, involving the removal of sequences that are tagged by OrthoDB as ‘uncharacterised’ or ‘hypothetical’. Also, any sequences with characters that do not fall into the standard amino acid codes were removed followed by filtering out redundant OrthoDB entries present in the sequence sets. In total, 131 431 sequences were retrieved for 40 proteins and post-processing removed 3383 sequences. The resulting ortholog sequences were mapped to AlphaFold Database and we obtained 54 924 AF predicted structures.

### Website framework and design

The database is launched using Apache HTTP server on Linux Platform. The object-oriented relational database management system Apache has been used to manage the retrieval and storage of data at back-end. The development of front-end website interface has been carried out using HTML, CSS, and JAVA scripts. The database significantly enhances access to information as full datasets are available for download.

#### Main page

The architecture of the Autophagy3D website is organized as a main home page listing 40 core autophagy proteins. The selection page of each protein is divided into three sections with extensive visualization components of protein structures.

#### Core structure page

The first default section displays the name of the protein followed by a general description of the protein, in particular its functional relevance in autophagy. This is followed by the sequence of the protein and the total structures available for the protein. Autophagy 3D facilitates the information regarding the availability of protein data bank (PDB) entries and AlphaFold predicted models. Their PDB IDs/AlphaFold IDs are displayed, and the structures are available for visualization and download. Additionally, a reference website hyperlink is also provided for source of the atomic coordinates.

#### Structure visualization

In the detailed structure page, Autophagy3D shows the three-dimensional visualization of the protein structures, showcasing detailed information about individual residues and chains. It offers various representations, including ball-and-stick, cartoon, spacefill, and Licorice. Users can customize these representations and visual styles, facilitating effective analysis and presentation of molecular structures. This section facilitates users to measure distances, angles, dihedral angles, and perform other analyses to explore molecular interactions and properties. Additionally, it allows users to download, take screenshots, and utilize a fullscreen viewer enhancing in-depth analysis. Interactive features such as zooming, rotating, panning, and selective component selection allow users to examine specific molecular structures closely. The implementation of the proposed structure section has been carried out using the WebGLviewer library for protein structure visualization [59]. It is a JavaScript library that provides interactive, hardware-accelerated 3-D representations of molecular data within web browsers. Utilizing WebGL (Web Graphics Library), the webpage efficiently visualizes molecular structures, offering a dynamic viewing experience. The 3D viewer is interactively connected with the sequence i.e. autophagy 3D enables users to inspect and navigate through individual residues within a loaded molecular structure using WebGL. It presents a graphical representation of these residues along with their associated information and functionalities. This functionality facilitates the user to interactively explore the details of the structure at the residue level, enabling detailed analysis, selection, and visualization of specific regions or residues of interest.

#### Interactor page

The interactor page in Autophagy 3D provides interactive visualization for various interactors associated with the protein. It showcases the graphical representation of the interactors of the protein displaying both experimental and AlphaFold structures. Within the web application, users are provided with the option to directly download the PDBs associated with the displayed structural information, thereby supporting further analysis or experimental investigations This visualization aids in understanding the associated interacting proteins, enriching the analysis of the protein’s interactions within cellular pathways. The interactome has been generated as a network using D3Blocks. D3Blocks is a D3 Javascript library used to create dynamic charts, where every node within the network is linked to its respective structural details.

#### Ortholog page

In addition, to structure and interactor web pages, an ortholog webpage is provided for each autophagy core protein, where metadata on orthologs such as UniProt ID, gene name, organism name, and protein length are displayed. Moreover, data related to the AlphaFold structure availability in the AlphaFold Database [38, 39], corresponding AlphaFold Database ID (if available), and overall pLDDT score are specified. The framework provides metadata available for download as CSV files and structural coordinate data as zip files.

#### Help page

A dedicated help page webpage has been incorporated into the website to provide assistance, guidance, and answers to commonly occurring issues users may encounter while using the database and 3D visualization of the protein. It also provides a brief about how the data has been extracted.

## Results

In humans, 40 core ATG proteins govern key events in the autophagy process, including autophagosome biogenesis, maturation and lysosome fusion. The core proteins include ULK1, ULK2, ULK3, ULK4, ATG2A, ATG2B, ATG4A, ATG4B, ATG4C, ATG4D, BECN1, BECN2, MAP1LC3A, MAP1LC3B, MAP1LC3B2, MAP1LC3C, GABARAP, GABARAPL1, GABARAPL2, ATG9A, ATG9B, ATG16L1, ATG16L2, RB1CC1, WIPI1, WIPI2, WIPI3, WIPI4, ATG3, ATG5, ATG7, ATG10, ATG12, ATG13, ATG14, PIK3R4, PIK3C3, ATG101, UVRAG and AMBRA1 [4–6]. For each protein, the database has curated structural information in three domains, namely, core structures, interactors, and orthologs (Fig. 1). The first information source is the experimentally derived structure, and in case the experimental structure was found unavailable, or the structural coverage of all PDB entries (of the core protein) was not greater than 70%, the corresponding AlphaFold structure was considered. Supplementary Figure S1 depicts a comprehensive workflow for data collection and more details are provided in the Materials and Methods section below.

**Figure 2.**
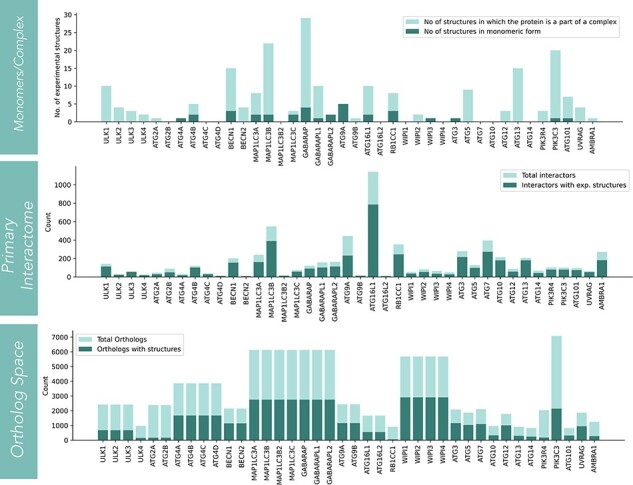
The statistics of structural coverage in Autophagy3D for the segments (such as core-autophagic proteins, interactome of the core proteins, and the corresponding ortholog structures) are shown in the series of bar graphs.

The statistics for three structural sections (core, interactors and orthologs) is shown in Fig. 2. In the core structure space, monomer and complex structures for each protein are displayed. The information on the number of experimental structures present for all 40 core proteins is shown in the first panel. Among the 40 core proteins, GABARAP has the highest number (28 in number) of structures present in complex with other proteins. Among monomers, PIK3C3 has the highest number of structures in monomeric form (16 in number). On the other hand, proteins like ATG2B, ATG4C/D, MAP1LC3B2, ATG16L2, WIPI1/4, ATG10 and ATG14 lack any experimental structures (low structural coverage). The second structural resource is the protein-protein interactome of each autophagic protein. Overall, there are 6377 interactors for 40 core autophagic proteins. The experimental structures have covered ∼70.4% of the total interactor space (4491), while AlphaFold models are available for 97.6% of proteins. The interactors that lacked AlphaFold models (2.4% of total interactors) were mostly pseudogenes and viral proteins. ATG16L1 has the largest number of interactors (1141) and followed by that, MAP1LC3B, ATG9A, ATG7 and RB1CC1 have relatively high protein-protein interactors. The third and final structural resource is orthologs. In this section, only structures from the AlphaFold database were considered. Among all proteins, PIK3C3 has the largest number of ortholog sequences (7082). However, the WIPI family of proteins has covered the structure space to a larger extent. For LC3 family of proteins (consisting of MAP1LC3A/B/B2/C, GABARAP, GABARAPL1/L2), PIK3C3, ATG4(A/B/C/D), ATG9 (ATG9A/9B), ATG3, BECN1/2, ATG7, ATG5 and ATG12, more than 1000 AF predicted models are available.

### Autophagy3D: a comprehensive web resource for autophagic protein structures

The https://autophagy3d.igib.res.in web server provides an intuitive interface for the autophagic community to access the structural database. The architecture of the website is organized as a main home page listing 40 core autophagy proteins. The selection page of each protein is divided into three sections with extensive visualization components of protein structures. The main page provides web browser-enabled visualization of structures that is crucial for examining three-dimensional macromolecules to achieve a detailed comprehension at the atomic level resolution thereby helping bridge the molecular details with the biological processes. Various standalone software packages for 3D structure visualizations are available [60–62], however, the software installation required. Recently JavaScript interpreters have been recommended to embed graphics applications directly within a web browser such as JSmol [63] and webGL [64]. RCSB has supported JSmol for the visualization of structures [65]. Autophagy3D utilises the WebGLviewer library for protein structure visualization.

**Figure 3.**
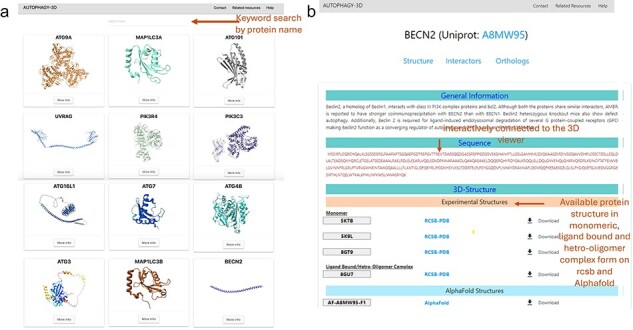
Structures of Autophagy3D database, where (a) shows the human autophagy proteins, where user can browse to human autophagy proteins using the browse button, (b) shows the available protein structures in monomeric, ligand bound and hetero-oligomer complex form on RCSB and AlphaFold database.

**Figure 4.**
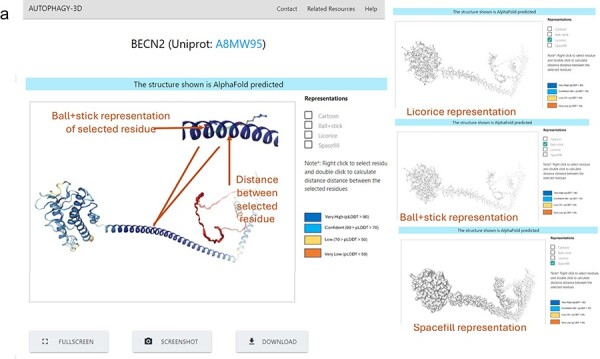
Protein Structure result in Autophagy3D database, where (a) shows 3D view of the protein structure, providing different representation, coloring based on pLDDT score, screenshot, fullscreen, and download options.

Figure 3 shows the demonstration of the web application interface where (A) displays a list of human proteins associated with the autophagy process, (B) displays available experimental protein structures in various forms such as monomer, peptide-bound, homo-oligomer, small molecule-bound, and hetero-oligomer from the RCSB database, as well as AlphaFold structures from the AlphaFold database. Users can view and download the respective structures, with reference hyperlinks provided for available structures. Further there is a section dedicated for sequence information of the protein, where each residue is interactively connected to the 3D viewer. Hence, autophagy 3D enables users to inspect and navigate through individual residues within a loaded molecular structure using WebGL. A detailed view of the core structure page is shown in Fig. 4. Upon hovering over a residue in the 3D visualization space, the residue name/position and atom ID are displayed. The visualization space has options to switch the representation styles to ball-and-stick, cartoon, licorice, and spacefill for a better understanding of the structure. In the case of AlphaFold structure, residues are colored according to pLDDT which reflects the residue-wise confidence score of the prediction.

**Figure 5.**
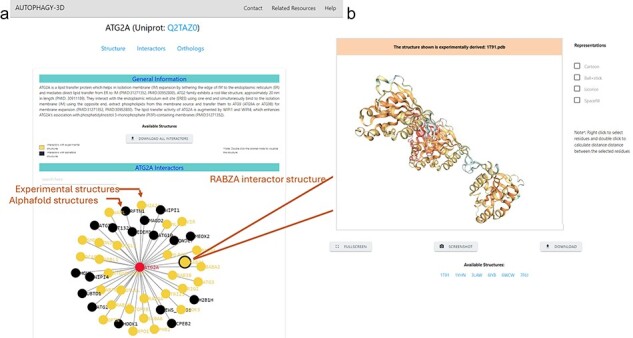
The interactome network is displayed on the webpage (a) The network is interactive and colored according to the availability of experimental structures, where on clicking a node (*i.e*. interactor), a new page gets opened on a new tab (b) shows the subpage of the interactor showcasing 3D-Visualisation space for the experimental structures of the interactor (AlphaFold model in case of non-availability).

Autophagy3D provides interactive visualization for interactors associated with the core proteins. These interactions are pivotal in numerous cellular processes and biological functions and are characterized experimentally. The page encompasses interactors obtained from the BioGrid 4.4 database. Interactors with experimental structure availability are provided in the database. Upon clicking a node in the interactive network, the 3D visualization of the respective node structure is executed where the available experimental structures can be visualized and downloaded (Fig. 5b). The header of the 3D visualization window has been colored showing experimental or AlphaFold predicted structure. This aids in understanding the associated interacting partners, further enriching the analysis of the protein interactions within cellular pathways. Figure 5 shows the interactome (of MAP1LC3B) in which nodes are colored according to the availability of the experimental structures in RCSB.

**Figure 6.**
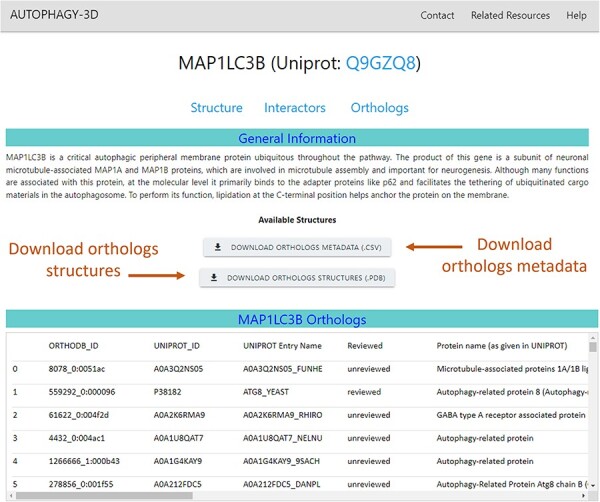
Orthologs in Autophagy3D database webpage displays MAP1LC3B metadata i.e. gene name, UniProt ID, sequence length, AlphaFold structure availability, pLDDT score etc., providing the facility to download the orthologs structures and its metadata.

The ortholog webpage of a core protein combines all the ortholog information obtained from OrthoDB and secondary annotation retrieved via mapping back to UniProt IDs. Along with this information, the page also provides structural data which are available for download for further analysis. The ortholog page (screen view shown in Fig. 6) presents ortholog metadata in tabular format. The list of orthologs belonging to core proteins is available for download in compressed PDB format. In addition, the associated metadata on the orthologs can also be downloaded as CSV files. The metadata includes gene name, respective UniProt/OrthoDB IDs, descriptions as given in UniProt and OrthoDB, Organism name, and length of the orthologous protein. Moreover, information regarding structural availability in the AlphaFold database, AlphaFold database ID, and average predicted local distance difference test (pLDDT) score of the protein are provided.

## Conclusions and future directions

Existing autophagy-specific databases provide information related to autophagy proteins [54], regulatory networks [55], expression profiles of autophagic proteins [56], miRNAs/lncRNAs associations [66, 67], post-translational modifications [68], small molecules targeting autophagic proteins [69–71] and LC3 interacting region (LIR) containing proteins [72]. To our knowledge, there exists no structural resource that is dedicated to structural information, and associated expansive knowledge, including interactors and orthologs of autophagy-related proteins. Autophagy3D, a web-based robust platform, offers extensive information on three-dimensional structures of autophagy-related human proteins. It offers comprehensive curated structural database of 40 core human autophagy proteins, including core structural data, protein-protein interactors, and orthologs. The structures are derived from experimental sources and recently available Alphafold2 predicted models. A total of 184 monomers/complex structures, 25 214 structures for interactors, and 54 924 structures for orthologs data have been made accessible, and the full dataset can be downloaded. The database will be regularly updated with new protein structural data, and we also plan to include AF2 multimer models of autophagic proteins with receptors, interactions with small molecules, and also extensive analysis on the evolutionary trajectory of proteins. In particular, orthologs can be studied in detail to explain heterogeneity in its functional role of autophagosome biogenesis. One way to investigate the functional activity is to study the evolution of its binding pockets. Understanding how these motifs are evolving is critical for understanding the development of new functionalities. Overall, Autophagy3D offers an interactive user-friendly design of the database that will facilitate easier access through its customizable settings and serve as a versatile information resource for researchers working in the field of autophagy.

## Supplementary Material

baae088_Supp

## Data Availability

The database significantly enhances access to information, as full datasets are available for download.
