# Supplementary material for: Autophagy3D: a comprehensive autophagy structure database
Source: Database (Oxford). 2024 Sep 19;2024:baae088. doi: 10.1093/database/baae088 (PMC11412239; doi:10.1093/database/baae088)
Supplement: baae088_Supp [file baae088_supp.zip › suppl_data/autophagy3D_SI_DATABASE.docx]

Supplementary information

Autophagy3D: a comprehensive autophagy structure database

Neha^1#^, Jesu Castin^1#^, Saman Fatihi^1,2^, Deepanshi Gahlot^1,2^, Akanksha Arun^1,2^ and Lipi Thukral^1,2*^

^1^ CSIR-Institute of Genomics and Integrative Biology, Mathura Road, New Delhi, 110025, India and

^2^Academy of Scientific and Innovative Research (AcSIR), Ghaziabad, 201002, India

^#^ Equal contribution.

^*^Corresponding author. [lipi.thukral@igib.res.in](mailto:lipi.thukral@igib.res.in)

| 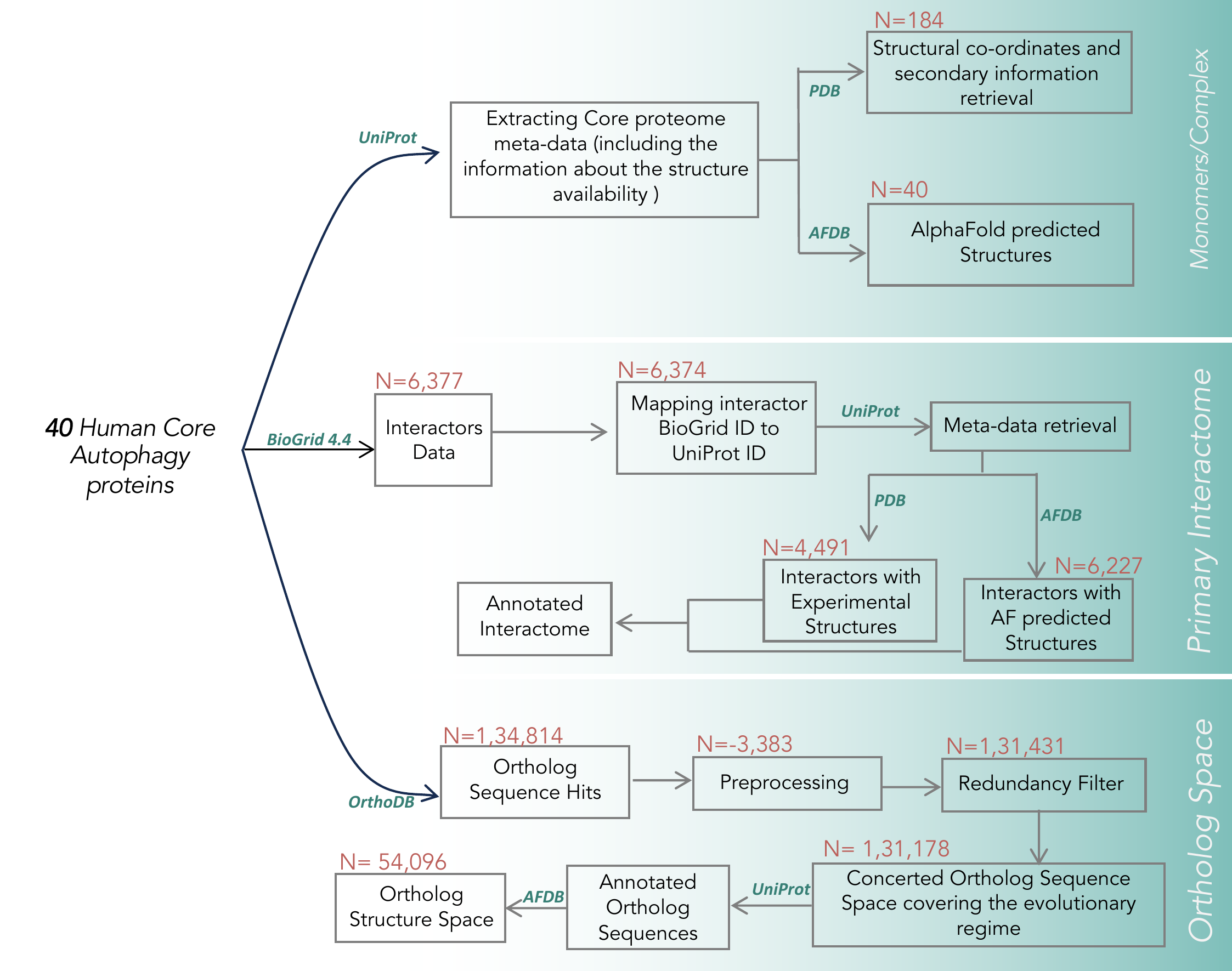 |
| --- |
| **Figure S1**. Pipeline for dataset collection/curation of Core Human Autophagy structures, primary interactors of the core proteins, and AlphaFold predicted models for orthologs. The amount of data retained at each step of the pipeline is mentioned along with the database utilized for curation.​ |
| **Alt text:** Detailed workflow of the dataset collection for Autophagy3D |
